# Glycated Hemoglobin and Methods for Its Point of Care Testing

**DOI:** 10.3390/bios11030070

**Published:** 2021-03-04

**Authors:** Miroslav Pohanka

**Affiliations:** Faculty of Military Health Sciences, University of Defense, Trebesska 1575, CZ-50001 Hradec Kralove, Czech Republic; miroslav.pohanka@gmail.com or miroslav.pohanka@unob.cz

**Keywords:** advanced glycation end products, analysis, bioanalysis, biosensor, chromatography, diabetes, diagnosis, glucose, hand held assay, lateral flow test, mass spectrometry

## Abstract

Glycated hemoglobin (HbA_1c_) is a product of the spontaneous reaction between hemoglobin and elevated glucose levels in the blood. It is included among the so-called advanced glycation end products, of which is the most important for the clinical diagnosis of diabetes mellitus, and it can serve as an alternative to glycemia measurement. Compared to the diagnosis of diabetes mellitus by glycemia, the HbA_1c_ level is less influenced by a short-term problem with diabetes compensation. Mass spectroscopy and chromatographic techniques are among the standard methods of HbA_1c_ level measurement. Compared to glycemia measurement, there is lack of simple methods for diabetes mellitus diagnosis by means of the HbA_1c_ assay using a point-of-care test. This review article is focused on the surveying of facts about HbA_1c_ and its importance in diabetes mellitus diagnosis, and surveying standard methods and new methods suitable for the HbA_1c_ assay under point-of-care conditions. Various bioassays and biosensors are mentioned and their specifications are discussed.

## 1. Introduction

Point-of-care testing has become a relevant part and aim of analytical and bioanalytical chemistry, and various target markers can be determined by these tests [1,2,3,4,5,6,7,8]. Although standard instrumental analyses, such as chromatography, mass spectrometric and electrophoretic analyses, have good potential to be used for the routine detection of biochemical, immunochemical and other markers, they are predetermined for laboratory use due to their complexity and costs. Simple methods for point-of-care diagnoses are available as well but they are typically suitable for simple markers and parameters (e.g., colorimetric clinical urine tests, electrochemical glucose tests). Some markers can be examined by colorimetric tests in the lateral flow immunochromatographic assay (e.g., pregnancy tests). In view of their complexity, many pathological processes and related diseases are not covered by adequate tests that are suitable for performance outside laboratories.

Glycated hemoglobin (HbA_1c_) is an additional marker, besides the standard glucose and glycemia analyses, that has become a relevant marker in new analytical methods. As discussed below, the determination of HbA_1c_ is substantial for diabetes diagnosis and provides substantial results compared to the simple measurement of glycemia [9,10,11]. Point-of-care testing of HbA_1c_ appears to be a suitable approach to timely and accurately revealing diabetes mellitus and it demonstrates a better quality of diagnosis compared to the standard determination of glycemia [12,13,14].

In this study, simple methods like biosensors and hand-held bioassays are reviewed and their practical relevance considering analytical parameters is discussed in the context of the standard analytical approaches. The analytical methods are discussed in view of their applicability in point-of-care testing. A survey of the current literature is provided as well.

## 2. Glycated Hemoglobin and Other Advanced Glycation End-Products

**Figure 1:**
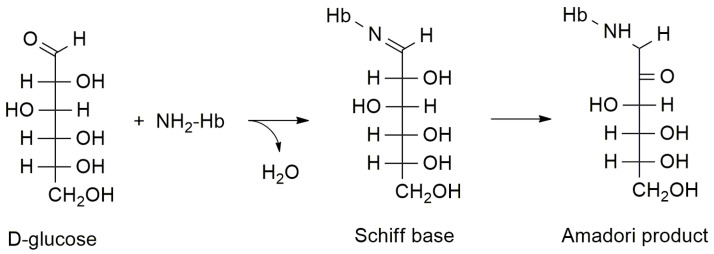
Chemical principles of hemoglobin glycation, Hb = hemoglobin.

HbA_1c_ is a glucose-modified hemoglobin created during the spontaneous reaction between glucose and N-terminal valine residues on β chains of hemoglobin-creating β-N-1-deoxy fructosyl [15]. The exact chemical mechanism of glycosylation is based on the formation of a Schiff base then shifting into rearrangement by means of Maillard reactions, eventually providing the final molecule with covalently bound glucose, called the Amadori product, or an advanced glycation end-product [16,17]. The principles of this chemical reaction are depicted in Figure 1. Once HbA_1c_ is formed, it remains in the blood circulation for quite a long time, typically from two to three months, because of the lifespan of erythrocytes, which is approximately 120 days [18]. The blood level of HbA_1c_ is quite stable and not sensitive to time of day, fasting or recently taken food [19]. All the aforementioned facts make HbA_1c_ a good marker for diabetes mellitus, with minimal misdiagnosis due to temporary and non-pathological changes in glycemia [20,21]. Though the measuring of HbA_1c_ is commonly considered a good way to diagnose diabetes mellitus, some pathologies like hemolytic anemia, which affects the lifespan of erythrocytes, or the presence of an abnormal chain in the hemoglobin molecule, can cause the distortion of results [22].

Hemoglobin is not the only protein providing advanced glycation end-products. The same mechanism happens for the other proteins located in the blood system, but their diagnostic meaning is less significant compared to the HbA_1c_. Glycated albumin may be mentioned as an important example. The blood or plasma level of glycated albumin is influenced by a time span of approximately two to three weeks [23], corresponding with the expected half-life of albumin, 15–20 days [18]. The glycosylation of albumin is dominantly made through lysine or less commonly by arginine [24]. The diagnostic meaning of glycated albumin is nearly the same as that of HbA_1c_ [25,26]. Although the glycated albumin level is not influenced by hemoglobin disorders, there can be changes in its blood concentration due to disorders in albumin metabolism like nephrotic syndrome, hyper- or hypothyroidism or liver cirrhosis [27]. When proteins become glycated, they also change in terms of their conformation and surface hydrophobicity compared to non-glycated structures [28,29,30]. The molecular weight of hemoglobin—64.5 kDa with one bound glucose at the most—can rise to 68 kDa when up to 15 glucose moieties are attached [31,32]. Fluorescence intensity can rise as well, from 34% for non-glycated hemoglobin up to 45% for HbA_1c_ [30]. Therefore, fluorescence can serve as an assay for the identification of hemoglobin types [33]. Raman spectroscopy can distinguish the types of hemoglobin as well [34]. The changes in surface hydrophobicity can be studied by reagents like 6-p-toluidinylnaphtalene-2-sulfonate and 8-anilinonaphtalene-1-sulfonate, providing fluorescence depending on the polarity of the solvent, developing low fluorescence in polar solvents (like water) and high fluorescence in low-polarity solvents [35,36]. Glycation of hemoglobin make HbA_1c_ less polar than the non-glycated hemoglobin, which can be visualized by 8-anilinonaphtalene-1-sulfonate [30]. Glycation of albumin leads to a slight increase in polarity, making it visible by the addition of a 6-p-toluidinylnaphtalene-2-sulfonate molecule [35]. The surface hydrophobic areas can serve for retention of the whole molecule during chromatographic isolation. For instance, hemoglobin was separated in a polar-phase system and showed a high value of the partition coefficient in a more hydrophobic environment such as polyethylene glycol polymer enriched with oleate [37]. Normal and elevated glycated hemoglobin were distinguished between phases composed of various amounts of PEG 600, Dextran 500 or polyvinylpyrrolidone. Researchers used polar phases and successfully distinguished levels of glycated hemoglobin indicated by the polar character of the surface [38,39].

The ratio of HbA_1c_ vs. the non-glycated hemoglobin serves for the diagnosis of diabetes mellitus. Healthy people have less than approximately 42 mmol/mol of HbA_1c_ compared to the total hemoglobin, representing 6.0%. Suspected diabetes mellitus (prediabetes) lies in the range of 42–47 mmol/mol, respectively, representing 6.0% to 6.4%. The presence of HbA_1c_ above the value of 48 mmol/mol, representing 6.5% and over, is typical for people suffering from diabetes mellitus [40,41]. The comparison of non-glycated hemoglobin and HbA_1c_ is presented in Table 1.

## 3. Standard Methods for Glycated Hemoglobin Assay

**Figure 2:**
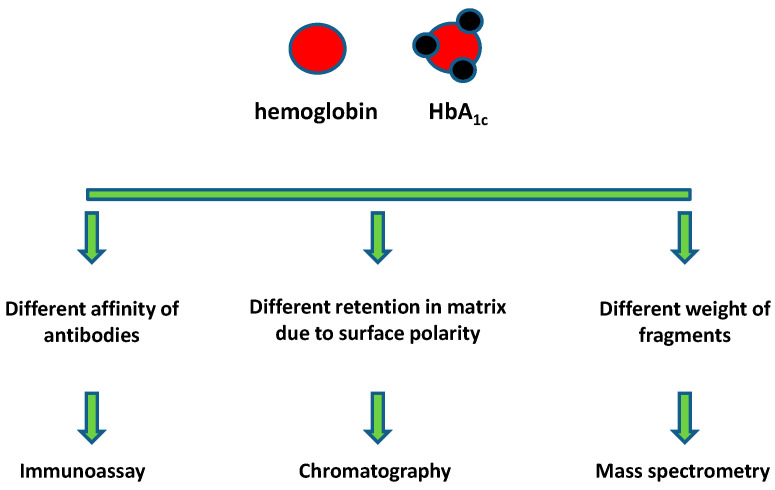
General principle of HbA_1c_ assay in the presence of standard hemoglobin.

Instrumental analytical methods serve as the standard tools for both recognizing new cases of diabetes mellitus and controlling whether the diagnosed diabetes mellitus is adequately compensated for [42]. In general, assays should be focused on distinguishing the standard hemoglobin and HbA_1c_. Physical and chemical differences between the two molecules serve the assay’s purpose. Interaction with antibodies creates the opportunity to distinguish the both types of hemoglobin by means of an immunoassay, different physical properties of the molecule surface (mainly due to surface polarity) allow isolation and determination by means of chromatography and the different weights of molecules and their fragments are the premise of mass spectrometry (MS). The general principles of the HbA_1c_ assay in the presence of standard hemoglobin are summarized in Figure 2.

Various chromatographic methods, spectrometric methods and their combination are common in the clinical praxis. High-performance liquid chromatography (HPLC) [43,44], cation exchange HPLC [45], Liquid chromatography (LC) tandem MS [46,47,48], matrix-assisted laser desorption ionization time-of-flight MS [49], capillary electrophoresis [50,51] and capillary zone electrophoresis tandem MS [52,53] can be mentioned as suitable for distinguishing between hemoglobin types. Immunochemical methods like the precipitation–turbidimetric method [50], fluorometric immunoassays [54] and the enzyme-linked immunosorbent assay (ELISA) [55,56,57] are also suitable for HbA_1c_ measurement.

The aforementioned instrumental analyses provide robust data about HbA_1c_ respectively to non-glycated hemoglobin. Though the analytical properties of the described methods are good enough to cover the expected ranges of HbA_1c_ compared to non-glycated hemoglobin, they are not suitable for performance outside equipped laboratories and their use requires educated laboratory staff. Apart from instrumental analyses, no fully applicable assay is available for point-of-care testing, despite the fact that such methods are highly desired and would improve the effectiveness of care for diabetes mellitus suffering patients. On the other hand, the instrumental analyses have become smaller and cheaper in recent years. Despite their limited application potential for point-of-care testing, better availability of instrumental analyses could be relevant for small laboratories, mobile hospitals, etc. Nevertheless, future research is expected to examine both directions: standard instrumental analyses and point-of-care tests.

## 4. Biosensors and Bioassays Measuring HbA_1c_

Handheld assays and tests, like various biosensors, hand-held bioassay test kits and similar analytical devices, could allow clinicians to make a diagnosis of diabetes mellitus in home conditions or conditions of small laboratories and private medical practices. They are not considered to be a replacement of the standard instrumental analytical methods, but biosensors and bioassays should be considered as a replenishment of the available set of methods, creating the opportunity to perform point-of-care tests. It is expected that biosensors and hand-held bioassays will be cheaper that the standard instrumental methods, will be applicable without expensive measuring or sample-processing devices and will require neither elaborative sample or reagents processing nor demands on staff training or education. Currently, there are methods and biosensors available for the rapid detection of glucose and glycemia level determination, and these devices exert good analytical parameters, simplicity and low costs, and noninvasive methods for measuring glucose have even been developed [58,59,60,61,62,63,64,65,66]. Though the methods for measuring glucose are promising and many of them are currently available in the market, they have limitations in the interpretation of glucose level, as discussed in the previous chapter.

**Figure 3:**
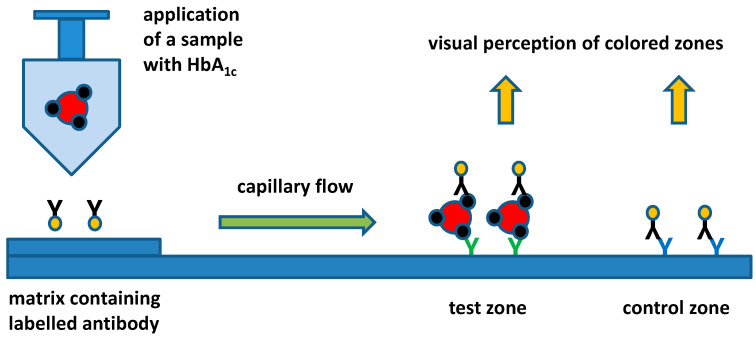
General principle of lateral flow test for HbA_1c_ assay.

Lateral flow immunochromatographic assays, also known as lateral flow tests, can be mentioned as a bioassay platform that would be applicable in point-of-care conditions. This assay works on the principle of analyte interaction with labeled (colored nanoparticles, fluorescence reagent, etc.) antibodies compared to other molecules exerting specific affinity. The analyte migrates by means of lateral flow and visible zones are formed by capturing either the analyte or the unreacted antibody by other recognition molecules (antibodies) that are immobilized on the thin-layer chromatography matrix. The general principle of the assay for HbA_1c_ is depicted in Figure 3. Various analytes including human chorionic gonadotropin (pregnancy test) and various antibodies and antigen markers can be measured by the lateral flow immunochromatographic assay and pregnancy tests can be mentioned as a common example of the actual use of these tests [67,68,69]. On the other hand, the lateral flow immunochromatographic assay provides a semiquantitative signal only and it is not fully applicable for the quantification of a marker, though there have been promising experiments aiming to make the assay suitable for the determination of exact concentrations [70]. The improved versions of lateral flow immunochromatographic assays can provide fully or partially quantified signals; on the other hand, instrumentation for color density, fluorescence intensity, Raman spectroscopy or other instrumentation is necessary in this case [71,72,73,74,75]. The use of instrumentation would make the lateral flow immunochromatographic assay more applicable for diabetes mellitus, but this also creates material demands on equipment and limits the ability to perform the lateral flow immunochromatographic assay in point-of-care conditions. In addition to the standard lateral flow tests, various microfluidic devices have become popular and applicable in practice [76,77,78,79].

In recent years, there has been great progress in the construction of biosensors and similar methods for the HbA_1c_ assay [80,81,82,83,84,85,86,87,88,89,90]. Various optical and electro-optical sensor methods have been developed in the past few years. A biosensor for the detection of HbA_1c_ was developed by Sun and coworkers using a surface plasmon resonance platform [91]. The researchers used an aptamer as the recognition part of the biosensor and were able to detect HbA_1c_ with a limit of detection of 2.55 nmol/L and a sensitivity of 1.06 × 10^−3^ RU/nmol/L. In another work, surface plasmon resonance with an immobilized aptamer served for the measurement of HbA_1c_ with a limit of detection of 1 nmol/L and a linear dynamic range of 18–147 nmol/L [92]. An aptamer was also used in the work by Lin and coworkers [93]. The authors immobilized the aptamer on a bacteriorhodopsin-embedded purple membrane as a physico-chemical transducer. The aptamer was specific against either HbA_1c_ or non-glycated hemoglobin. The interaction of aptamer with the HbA_1c_ or non-glycated hemoglobin reduced the detected photocurrent because of partial light absorption by the captured analyte. The assay exerted equal limits of detection for both types, under 0.1 μg/mL, and a dynamic range of 0.1–100 μg/mL in a 15-min measuring cycle. An electrochemiluminescence sensor was constructed for the measuring of HbA_1c_ using Tris(2,2′-bipyridyl)dichlororuthenium(II)-doped mesoporous polydopamine nanoparticles covered with an aptamer specific to HbA_1c_ [94]. Interaction of the prepared nanoparticles with HbA_1c_ caused quenching of ruthenium complex electrochemiluminescence. The authors declared the limit of detection to be 0.015% HbA_1c_ from the total hemoglobin, and the linear range was 0.1–18.5%. Further improvements in optical and electro-optical analytical devices may be based on colorimetric plasmonic sensors [95,96,97].

Bioanalytical methods and biosensors can work on voltametric principles, as seen in the following cited papers. Shahbazmohammadi and coworkers immobilized fructosyl peptide oxidase with graphene oxide and gold nanoparticles on working electrodes [98]. Fructosyl valyl histidine served as a mimetic of HbA_1c_ and was oxidized by the immobilized enzyme. The amperometric detection provided response in the calibration range 0.1 to 2 mmol/L with a limit of detection for fructosyl valyl histidine of 0.3 μmol/L. In another work, fructosyl amine oxidase immobilized on gold and platinum composite nanoparticles served for HbA_1c_ oxidation and amperometric detection [85]. In another work, a piezoelectric quartz crystal microbalance biosensor was made using iron oxide nanoparticles and a polyclonal antibody specific to HbA_1c_ [99]. The oscillation frequency of the biosensor dropped when HbA_1c_ was caught by the immobilized antibody. The assay exerted a limit of detection of 0.045 mg/mL and it fully correlated to the standard ELISA. The fact that the assay can be finalized in a single step, consisting of the sample application, is a major advantage. A voltametric biosensor-based graphite sheet electrode was constructed by Jaberi and coworkers [100]. The researchers covered the graphite sheet with a nanocomposite composed of reduced graphene oxide and gold and further with a DNA aptamer specific to HbA_1c_. The interaction with HbA_1c_ caused a change in voltametric sensitivity to Prussian blue presented in the ambient solution, and differential pulse voltammetry served for the response measurement. The biosensor had a linear range of 1 nmol/L–13.8 μmol/L, a sensitivity of 269 μA/cm^2^ and a limit of detection of 1 nmol/L for the HbA_1c_ assay.

Affinity interactions with HbA_1c_ can be based on simpler molecules than the aforementioned antibodies and aptamers. Derivatives of boronic acid appear to be suitable reagents for this interaction [101,102,103,104]. An electrochemical sensor system for HbA_1c_ detection using boronic acid was proposed in the work of Wang and coworkers [105]. The researchers prepared gold nano-flowers modified by 4-mercaptophenylboronic acid and the whole complex was located on graphite screen-printed electrodes. HbA_1c_ was caught on the 4-mercaptophenylboronic acid and then catalyzed the reduction of hydrogen peroxide, which was recorded by cyclic voltammetry. Gold nanoflowers improved the transport of electrons from the reaction to the electrode. The assay exerted a linear dynamic range of 5–1000 μg/mL representing 2–20% of HbA_1c_ for an assay lasting 65 min. Boronic acid can serve as a matrix for the imprinting of HbA_1c_ relative to non-glycated hemoglobin and for making a molecularly-imprinted polymer, as described in the work by Pandey and coworkers [106]. A molecularly imprinted polymer was made from aminophenylboronic acid with poly-rhodamine b nanocubes and deposited on carbon paste-coated aluminum foil by electropolymerization. The interaction of HbA_1c_ (relative to non-glycated hemoglobin) with the sensor changed the voltametric properties of the electrode, which was measured. The sensor provided a limit of detection equal to 0.08 ng/mL for the non-glycated hemoglobin and 0.09 ng/mL for the HbA_1c_. A survey of selected aforementioned methods is presented in Table 2.

Real point-of-care assays for the detection HbA_1c_ by small portable devices appear to be a possibility in the coming years. The current research on biosensors and similar bioassays appears to be promising. Even though many of the assays proposed in the literature are not suitable for mass commercial production because their parts (specific nanoparticles or handmade aptamers, for instance) are not available in the market, this situation may change in the future. Research and development on HbA_1c_ point-of-care tests can be further intensified when their marketing is supported by health insurance companies, as with standard glucose tests, which are provided or paid out to diabetic patients in some countries.

## 5. Conclusions

Glycated hemoglobin is an important biochemical marker that provides more reliable clues for diabetes mellitus diagnosis than glucose and glycemia measurements. Compared to the glucose assay, the point-of-care determination of HbA_1c_ has not been successfully commercialized and new measuring devices are being extensively investigated. The practical impact of the current research is expected to be seen in the future, when the point-of-care assays for HbA_1c_ may become a relevant analytical tool, making the accurate diagnosis of diabetes mellitus more available in future clinical practice. Future research should be focused on the development of simple methods for HbA_1c_ quantitative assays based on portable detectors.

## Figures and Tables

**Table 1:** Basic specifications of non-glycated hemoglobin and HbA_1c_.

Specification	Non-Glycated Hemoglobin	HbA_1c_	References
Number of glycated sites/molecular weight	1/64.5 kDa	15/68 kDa	[31,32]
Florescence intensity	34%	45%	[30]
Hydrophobicity	high	low	[38,39]
Percentage in blood of health people	above 94%	under 6.0%	[40,41]
Percentage in blood of people with prediabetes	94.0–93.5%	6.0–6.5%	[40,41]
Percentage in blood of people with diabetes mellitus	under 93.5%	above 6.5%	[40,41]

**Table 2:** Biosensors and bioassays for HbA_1c_ measurement.

Principle of Assay	Recognition Parts in the Assay	Specifications	Limit of Detection	References
Surface plasmon resonance	aptamer	sensitivity 1.06 × 10^−3^ RU/nmol/L	limit of detection 2.55 nmol/L	[91]
Surface plasmon resonance	aptamer	linear dynamic range 18–147 nmol/L	limit of detection 1 nmol/L	[92]
Measuring of photocurrent using bacteriorhodopsin and aptamer embedded membrane, interaction with analyte causes reduction of photocurrent	aptamer	dynamic range 0.1–100 μg/mL in a 15 min measuring cycle	limit of detection under 0.1 μg/mL	[93]
Quenching of ruthenium complex containing nanoparticles electrochemiluminescence in the presence of HbA_1c_	aptamer	linear range 0.1–18.5%	limit of detection 0.015% HbA_1c_ from the total hemoglobin	[94]
Enzyme catalyzed oxidation of fructosyl valyl histidine as a mimetic of HbA_1c_, amperometric detection followed	fructosyl peptide oxidase	calibration range 0.1 to 2 mmol/L	limit of detection 0.3 μmol/L	[98]
Quartz crystal microbalance biosensor with immobilized antibody directly interacted with HbA_1c_, drop in oscillation frequency followed	polyclonal antibody	-	limit of detection 0.045 mg/mL	[99]
Voltametric biosensor with immobilized aptamer, interaction with HbA_1c_ caused change in sensitivity to Prussian blue in ambient solution	aptamer	linear range 1 nmol/L–13.8 μmol/L, sensitivity 269 μA/cm^2^	limit of detection 1 nmol/L	[100]
HbA_1c_ was caught by boronic acid and then catalyzed reduction of hydrogen peroxide, which was recorded by cyclic voltammetry	gold nanoparticles covered with 4-mercaptophenylboronic acid	linear dynamic range 5–1000 μg/mL respective 2–20%, assay lasting 65 min	-	[105]
Interaction of non-glycated hemoglobin respective to HbA_1c_ with molecularly imprinted polymer caused change in voltametric characteristics	molecularly imprinted polymer based on boronic acid	-	limit of detection equal 0.08 ng/mL for the non-glycated hemoglobin, 0.09 ng/mL for the HbA_1c_	[106]

## Data Availability

All data are provided in this work.
